# The Outcomes and Quality of Pancreatic Islet Cells Isolated from Surgical Specimens for Research on Diabetes Mellitus

**DOI:** 10.3390/cells11152335

**Published:** 2022-07-29

**Authors:** Ju Yun Oh, Yang Hee Kim, Song Lee, Yu Na Lee, Han Se Go, Dae Wook Hwang, Ki Byung Song, Jae Hoon Lee, Woohyung Lee, Seongjun So, Eunju Kang, Eunsung Jun, In Kyong Shim, Song Cheol Kim

**Affiliations:** 1 Asan Institute for Life Science, University of Ulsan College of Medicine and Asan Medical Center, 88 Olympic-ro 43-gil, Songpa-gu, Seoul 05505, Korea; xlsk93@naver.com (J.Y.O.); yh100828@hanmail.net (Y.H.K.); ssong277@hanmail.net (S.L.); ynlee426@naver.com (Y.N.L.); standard0130@gmail.com (H.S.G.); soseongjun7@gmail.com (S.S.); ekang@chamc.co.kr (E.K.); go1040@hanmail.net (E.J.); 2 Division of Hepato-Biliary and Pancreatic Surgery, Department of Surgery, University of Ulsan College of Medicine and Asan Medical Center, 88 Olympic-ro 43-gil, Songpa-gu, Seoul 05505, Korea; dwhwang@amc.seoul.kr (D.W.H.); mtsong21c@naver.com (K.B.S.); hbpsurgeon@gmail.com (J.H.L.); ywhnet@gmail.com (W.L.); 3 Asan Medical Institute of Convergence Science and Technology (AMIST), University of Ulsan College of Medicine and Asan Medical Center, 88 Olympic-ro 43-gil, Songpa-gu, Seoul 05505, Korea; 4 Department of Convergence Medicine, University of Ulsan College of Medicine and Asan Medical Center, 88 Olympic-ro 43-gil, Songpa-gu, Seoul 05505, Korea

**Keywords:** pancreatic islet, human islet isolation, diabetes, partial pancreas, surgical specimens

## Abstract

Isolating a large quantity of high-quality human islets is a prerequisite for diabetes research. Human islets are typically isolated from the pancreases of brain-dead donors, making research difficult due to low availability. Pancreas tissue discarded after surgical resection may be a good alternative source of islet cells. To test this hypothesis, we isolated islets from discarded surgical specimens and evaluated the islet yield and quality as well as islet cell preparations. Eighty-two segmental pancreases were processed using the Ricordi automated method, and islet yield and quality were investigated. The mean age of patients was 54.6, and the cohort included 32 diabetes patients. After purification, partially resected pancreases yielded an average of 59,593 ± 56,651 islet equivalents (IEQs) and 2546 IEQ/g of digested pancreas, with 71.5 ± 21% purity. Multivariate analysis revealed that diabetes (*p* = 0.0046) and the lobe used (*p* = 0.0156) significantly altered islet yield. Islets transplanted into diabetic mice displayed good viability and in vitro glucose responses, DNA/RNA quality, mitochondrial function, and glucose control, even though these results were dependent on islet quality. Isolated cells also maintained high viability and function even after cryopreservation. Our findings indicate that pancreatic tissue discarded after surgery can be a valuable source of islets for diabetes research.

## 1. Introduction

Human pancreatic tissue and isolated islets of Langerhans cells are vital resources for diabetes research [1]. Human islets have been used to study islet morphology, genomics, insulin and glucagon secretion, transcription factor regulation, transplantation, and many other aspects of endocrine physiology and diabetes [2]. The pancreas of large mammals differs considerably from that of rodents. The structure, size, and shape of the islets and their attachment to the surrounding exocrine tissue are only some of the variables that make islet isolation unique and different for each species. Even within a species, there can be significant differences depending on the age, volume, and condition of the exocrine pancreas that affect the outcome of the islet isolation procedure. The isolation of islets from the human pancreas is a difficult and sensitive process that requires a specialized and licensed facility, as well as the combined efforts of multiple trained personnel. Although human islet isolation techniques have constantly been improving, it is still difficult to recover a sufficient number of islets from a single donor pancreas [3,4]. Several studies have identified donor-, pancreas-, and isolation-related factors that influence islet yields [2,5,6]. In our previous study, we described our experiences with isolating islets from human cadaveric donors and our analysis of preexisting donor factors and isolation variables that may affect the results of human islet isolation [7].

Human islets are typically isolated from the pancreas of a cadaveric donor, which makes research on islet cells for diabetes treatment difficult as islet availability is often low, particularly in countries that prohibit the use of organs procured from cadaveric donors for research use. Regardless, islet availability is often low, even in countries that accept the use of such organs for research. While pancreatic islets can also be derived from live donors who have undergone a partial pancreatectomy, it has been rarely reported in the literature, and these studies did not include qualified data [8,9,10,11]. As noted above, the variability of conditions associated with donors and the pancreas tissue itself also makes the consistently successful isolation of islets difficult, especially under surgical conditions. The aim of this study was, therefore, to evaluate the use of surgical specimens from pancreatectomized patients as an alternative source of human islet tissue by examining the quantity and quality of the isolated islets. We evaluated the quality of islet samples isolated from pancreases discarded after operations. Our data suggest that islets can be effectively purified from partial pancreatectomy specimens; in addition, the quality of the isolated human islets can be maintained in both in vitro culture and in vivo transplantation assays.

## 2. Materials and Methods

### 2.1. Pancreatic Resection and Harvest of Pancreatic Tissue

The study cohort comprised patients with pancreatic disease who were scheduled for pancreatic resection. All patients provided fully informed consent to participate in this study. This study was approved by the Institution Review Board of Asan Medical Center, Korea (IRB number: 2017-0503). Pancreases were resected according to the standard surgical resection method and removed from the body cavity. Pancreas specimens were examined for pathologic lesions by a scientist, and a portion of the tissue irrelevant to disease diagnosis was excised for islet isolation. The pancreatic duct was cannulated with an angio-catheter and fixed to the pancreas for future collagenase infusion in an operating room with sterile facilities. One to two milliliters of cold histidine-buffered tryptophan ketoglutarate (HTK) solution (Dupont Pharm, Wilmington, NC, USA) was injected before transportation to the isolation laboratory. Pancreatic tissues were kept in an iced preservation solution during transfer to the laboratory.

### 2.2. Islet Isolation

**Figure 1:**
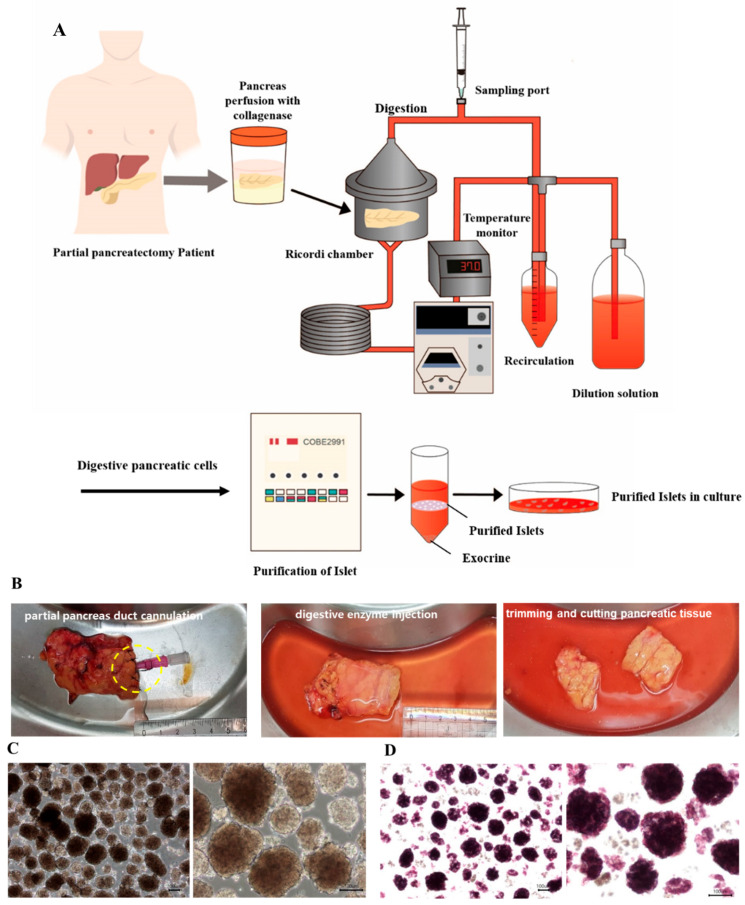
Isolation of islet cells through partial tissue acquisition, digestion, and purification. (**A**) Diagram of the islet isolation process. (**B**) Digestive enzyme was injected via the pancreatic duct exposed on the cross-section of the cut pancreatic tissue. Considerable swelling of the tissue was taken as confirmation that the digestive enzyme had effectively entered the entire pancreas. The pancreas was then cut to a size suitable for mechanical dissociation in the chamber. (**C**) The morphology of the isolated islet cells; scale bar: 100 µm. (**D**) The identification of pure islet cells stained with dithizone; scale bar: 100 µm. Yellow circle: is a picture using angiocath to inject collagenase into the duct.

Islets were isolated using an automated method described initially by Ricordi et al. and also in our previous report (Figure 1) [5]. Before the enzyme was injected into the partial pancreas, the fat was cut, and the tissue was trimmed. The enzyme used for isolation was either Liberase MTF C/T (collagenase 0.5 g/350 mL and thermolysin 0.015 g/350 mL) (Roche Diagnostics, Roche Applied Science, Indianapolis, IN, USA) or Collagenase P (0.18 g/200 mL; Sigma-Aldrich, MO, USA), reconstituted in Hank’s Balanced Salt Solution (HBSS) (Sigma-Aldrich). The digestion of the pancreas was performed using mechanical dissociation and enzymatic recirculation. Islet cells were identified by dithizone (DTZ) (Sigma-Aldrich) staining. Islet cells were purified using a COBE2991 processor (Cobe BCT, Lakewood, CO, USA) and OptiPrep™ density gradient medium (Sigma-Aldrich).

### 2.3. Islet Yield and Viability

Islet cells were stained with the zinc chelator DTZ. DTZ (100 mg) was first dissolved in 10 mL of dimethyl sulfoxide (DMSO) and then diluted with 20 mL of HBSS. After filtration through a 0.22 μm syringe filter, the diluted DTZ solution was used to stain islets. Islet yields were measured and recorded as islet equivalents (IEQs) at both the pre-purification and post-purification stages. Islet yields were normalized per gram of pancreatic tissue by dividing the total IEQ by the pancreas weight (g) prior to digestion.

### 2.4. Islet Culture

Islet preparations were maintained in standard 100 mm non-coated dishes, each containing 15 mL of CMRL1066 (Gibco, NY, USA) standard culture medium supplemented with 10% fetal bovine serum (FBS) (Gibco), 2 mM Glutamax (Gibco), and 1% antibiotic-antimycotic (Gibco). Islets were maintained in an incubator at 37 °C with a 5% CO_2_ atmosphere for 24 and 72 h. Islets were aliquoted at 10,000 IEQ per dish.

### 2.5. Glucose-Stimulated Insulin Secretion (GSIS) Assay

To measure GSIS, isolated islets were starved for 1 h in a Krebs–Ringer buffer at 37 °C. We then replaced the medium with a low-glucose (2 mM) Krebs–Ringer buffer and incubated the cultures for 1 h, collected the supernatants, and replaced the medium with a high-glucose (20 mM) Krebs–Ringer buffer. After a further 1 h of incubation, we collected the supernatants and analyzed the concentration of released insulin using a Human Insulin ELISA kit (American Laboratory Products Company (ALPCO), Salem, MA, USA).

### 2.6. RNA and DNA Extraction

RNA was extracted from isolated pancreatic islet cells using the commercially available RNeasy^®^ Mini Kit (Qiagen GmbH, Hilden, Germany). Genomic DNA was extracted using the DNEasy Blood and Tissue DNA isolation kit (QIAGEN). Manufacturer protocols were followed in both cases.

### 2.7. Immunostaining

Islets were fixed with 4% paraformaldehyde (PFA) (Merck, Darmstadt, Germany), embedded in paraffin, and serially sectioned (4 µm sections). The islet paraffin sections were stained with hematoxylin and eosin and subjected to histological analysis using a microscope. For immunofluorescence staining, islets were cultured on 100 mm non-coated dishes, fixed with 4% PFA for 30 min at 4 °C, then washed three times with phosphate-buffered saline (PBS). Cells were permeabilized with 0.5% Triton X-100 at 4 °C for 5 min and washed three times with PBS. To block the nonspecific binding of the antibody, cells were incubated in 5% normal goat serum or normal horse serum for 30 min at room temperature. The primary antibodies used were mouse anti-insulin (1:50; Santa Cruz Biotechnology, Santa Cruz, CA, USA) and rabbit anti-glucagon (1:50; Santa Cruz Biotechnology). Membranes were incubated in primary antibodies overnight at 4 °C. For secondary antibody fluorescence labeling, cells were incubated with Alexa Fluor 488 goat anti-mouse or anti-rabbit IgG antibody (1:200; Life Technologies, Carlsbad, CA, USA). Hoechst 33,342 (1:100; Thermo Scientific, Rockford, IL, USA) was used to stain nuclei for 3 min at room temperature, and cells were then washed three times with PBS. The slides were visualized under an LSM710 confocal microscope (Carl Zeiss, Oberkochen, Germany).

To confirm the immunohistochemistry results regarding the expression of insulin and glucagon, the islet cell kidney graft was fixed in 4% PFA for 30 min at 4 °C and washed twice with PBS on day 40. The islet cell kidney transplant was embedded in Tissue-Tek (Sakura Finetek, Torrance, CA, USA) and sectioned (6 μm) to acquire frozen tissue blocks. The cells were permeabilized with 0.1% Triton X-100 at 25 °C for 10 min and washed thrice with PBS. For antibody blocking, the cells were incubated in 3% bovine serum albumin for 1 h at 25 °C. The primary antibodies used were mouse anti-insulin (1:50; Santa Cruz Biotechnology, Santa Cruz, CA, USA) and rabbit anti-glucagon (1:50; Santa Cruz Biotechnology). Membranes were incubated in primary antibodies overnight at 4 °C. For secondary antibody fluorescence labeling, cells were incubated with either Alexa Fluor 488 goat anti-mouse or anti-rabbit IgG antibody (1:200; Life Technologies, Carlsbad, CA, USA). Hoechst 33,342 (1:100; Thermo Scientific, Rockford, IL, USA) was used to stain the nuclei for 3 min at room temperature, and the cells were then washed thrice with PBS. The slides were visualized under an LSM710 confocal microscope (Carl Zeiss, Oberkochen, Germany).

### 2.8. Mitochondrial Respiratory Function Assessment

Islet cells were dissociated using TrypLE (Gibco) for 5 min. Dissociated islet clusters were seeded on a 24-well seahorse cell culture plate (Seahorse Biosciences, North Billerica, MA, USA) at a concentration of 50 clusters per well. Mitochondrial respiratory function was measured using an XF Cell MitoStress Test kit in an XF24 Extracellular Flux analyzer (Seahorse Biosciences), as described previously [12,13]. The mitochondrial oxygen consumption rate (OCR) was measured by serial treatment with oligomycin (1.5 µM) for ATP production (oligomycin OCR–basal OCR), carbonyl cyanide 4-(trifluoromethoxy) phenylhydrazone (FCCP) (1 µM) for maximal respiration and reserve capacity (maximal OCR–basal OCR), and antimycin A (0.5 µM) and rotenone (0.5 µM) for non-mitochondrial oxygen utilization. Oxygen consumption was normalized to baseline oxygen consumption by measuring total DNA levels.

### 2.9. Cryopreservation and Thawing

Islet cells (1500 IEQ) were resuspended in a cryopreservation medium (90% FBS/10% DMSO) and cryopreserved using a CryoMed controlled-rate freezer (Thermo Fisher Scientific Inc., Waltham, MA, USA, Non-IVF 7452). The program used was as follows: start temperature of 0 °C, 0.2 °C/min to −40 °C, and −25 °C/min to −150 °C. Cryovials were then transferred to liquid nitrogen.

Cryopreserved islet cells were rapidly thawed in a 37 °C water bath. The thawed cells were diluted in 10 mL of culture medium and centrifuged at 280× *g*. After removing the supernatant, islet cells were resuspended in a standard culture medium and incubated for 24–48 h at 37 °C and 5% CO_2_. Thawed islet cells were analyzed for viability using fluorescence diacetate (FDA) (0.5 µM; Sigma-Aldrich, MO, USA) and propidium iodide (PI) (75 µM; Sigma). Viability was determined by calculating the ratio of viable FDA-positive cells (green) to non-viable PI-positive cells (red). We compared the conventional protocol using an isopropanol-based freezing container (IFC) with the controlled-rate freezer (CRF) protocol. Samples in both groups (triplicate samples of the same donor for three patients) contained 1500 IEQ and were frozen in the same cryopreservation medium.

### 2.10. Transplantation of Islets into Diabetic Mice

Male 8-week-old BALB/c nude mice were used for in vivo assessments of islet viability (*n* = 5). The animals were cared for in accordance with the Guidelines for Laboratory Animal Care of Asan Medical Center. Diabetes was induced by a single intraperitoneal injection of streptozotocin (STZ) (180 mg/kg; Sigma Chemical, St. Louis, MO, USA). Diabetic mice with non-fasting blood glucose values > 350 mg/dL, as measured with a blood glucose monitor (Accu-Check; Roche, Applied Science, Indianapolis, IN, USA) for more than 3–4 consecutive days, were used as recipients of islet grafts. To carry out a transplantation assay, 2000 IEQ of human islets were transplanted under the kidney capsule of the diabetic mice. The cells were transferred through PE50 tubing. After anesthetizing each mouse with isoflurane, we exposed the kidney and slowly injected the cells into the tubing under the kidney capsule using a Hamilton syringe. After removing the tubing, the nick in the kidney capsule was carefully closed, and the kidney was gently replaced inside the peritoneum. Blood samples were drawn daily from the tail vein of recipients for the first five days of observation and every second day for the next 87 days of monitoring. Postprandial serum glucose levels were determined using the Accu-Check glucose analyzer. Non-fasting serum glucose levels < 200 mg/dL were defined as normoglycemia and considered to represent a functional graft. After 90 days of observation, a nephrectomy of the graft-bearing kidneys was performed to demonstrate an immediate return of hyperglycemia.

### 2.11. Intraperitoneal Glucose Tolerance Test (IPGTT)

Mice were made to fast for 6 h during glucose and insulin tolerance tests as well as glucose-stimulated insulin secretion tests. After that, they were injected with D-glucose solution (2 g/kg) via intraperitoneal injection (IP). In addition, the mouse blood glucose level was recorded at selected intervals after the IP. Blood samples were kept on ice during collection and centrifuged at 450× *g* for 10 min at 4 °C, and the obtained plasma was stored at −20 °C. Plasma samples were analyzed using an ultrasensitive C-peptide ELISA kit (Mercodia, Uppsala, Sweden). Measurements were performed on a spark plate reader (TECAN Group Ltd., Männedorf, Switzerland) and analyzed using Prism8 software (GraphPad Software Inc., San Diego, CA, USA.)

### 2.12. Statistical Analysis

Statistical tests performed for specific data sets are described in the corresponding figure legends. Two-tailed unpaired *t*-tests (Student’s *t*-test) were used to measure standard deviation (SD). A two-way ANOVA test for multiple comparisons was used to calculate significance, including *p*-values. A *p*-value < 0.05 was considered to be a significant difference. Linear regression analysis was performed to identify the factors associated with the outcomes of islet cell isolations. A logarithmic transformation was performed to normalize skewed variables. Variables were selected using stepwise selection in multiple regression analyses.

## 3. Results

### 3.1. Donor Characteristics

The condition of the pancreatic tissue is important for effectively isolating islet cells from a partial pancreas. Table 1 summarizes the donor demographics, including the age, sex, body/mass index (BMI), presence of diabetes, and the location and size of the partial pancreas used for islet isolation. A total of 82 patients donated tissue for islet cell isolation. The average age of the donors was 54.6 ± 15.07 years, and the cohort comprised 52.8% females and 47.13% males. The average BMI was 25.5% ± 8.56. Of the patients who donated pancreatic tissue, 20%, 6.36%, and 10% underwent the surgical resection of localized neuroendocrine tumors (NETs), benign intraductal papillary mucinous neoplasms (IPMNs), and solitary pseudopapillary neoplasms (SPNs), respectively. Of the patient tissues used for islet cell isolation, 57.8% of the samples were from patients without diabetes, and 42.2% were from patients with diabetes. Of the pancreatic specimens used for islet isolation, 26.2% were from the head and 73.8% from the body and tail of the organ. The average time from the operating room to the laboratory was 31.1 min. Patient information used for islet cell function analysis is presented in the Supplemental Data (Table S1).

### 3.2. Islet Yield from the Partially Resected Pancreas

The average weight of the specimens was 23.4 ± 10.6 g, which was one-quarter of the size of the entire pancreas. Of the specimens, 50.6% were digested with Collagenase P and 49.4% with Liberase MTF/CT, with an average digestion time of 10.47 ± 5.8 min. The IEQ values before and after purification were 124,006 ± 88,959 and 59,593 ± 56,651, respectively. The purity of the purified islets was 71.5 ± 21.0% on average.

### 3.3. Factor Analysis of Isolation Outcomes

Using multivariate analysis, we analyzed whether gender, age, the presence or absence of diabetes, BMI, enzyme type, or digestion time affected the yield of islet cells during the isolation process; this analysis was due to the diversity of characteristics associated with partial pancreas donors. The presence of diabetes and the location of the pancreas specimens were independent factors affecting islet yield before and after purification (Table 2). There was a significant difference between patients without and with diabetes both before (6588.86 vs. 3712.82 IEQ/g) and after purification (3088.35 vs. 1655.95 IEQ/g, *p* < 0.05). However, it was not related to the duration of diabetes (Figure S1). The yield isolated from the tail portion was also significantly higher than the yield from the head portion (6364.14 vs. 3063.71 IEQ/g, *p* < 0.00001) (Figure S1).

### 3.4. DNA and RNA Quality, Glucose Responsiveness, and Histology

**Figure 2:**
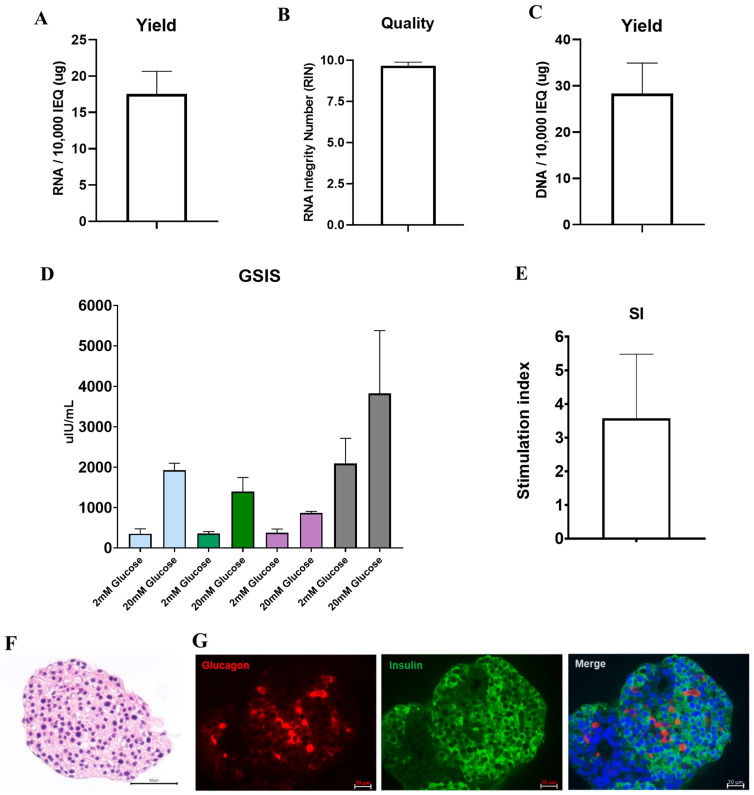
Quality and function of islet cells isolated from partial pancreas tissue. (**A**) The amount of RNA, (**B**) RNA integrity number (RIN) value, and (**C**) amount of DNA extracted from 10,000 islet equivalents (IEQs) (*n* = 16). (**D**) The concentration of insulin secreted at glucose stimulation concentrations of 2 mM and 20 mM, and (**E**) the insulin index of the difference between the 2 mM and 20 mM glucose concentrations (quadruplicate in 4 patients). (**F**) The morphology of the isolated islet cells was observed using hematoxylin and eosin staining (scale bar: 50 µm) and (**G**) the expression of glucagon and insulin was confirmed by immunofluorescence staining (scale bar: 20 µm). GSIS, Glucose-stimulated insulin secretion; SI, Stimulated index.

We performed RNA and DNA extraction and GSIS assays on the third day of culture to examine the quality and functional integrity of islet cells isolated from partial pancreas tissue. An average of 17.58 ± 1.77 μg of RNA was obtained from 10,000 IEQ of islet cells (Figure 2A). The RNA integrity number (RIN) value, a measure of RNA quality, was 9.6, indicating that high-quality RNA could be extracted from the cells (Figure 2B). DNA extraction yielded an average of 22.00 ± 4.47 μg per 10,000 IEQ (Figure 2C). Both RNA and DNA yields were satisfactory for use in future experiments. In addition, the islet cells were also evaluated for normal insulin secretion in response to glucose using the GSIS assay. The average stimulation index was 3.85 ± 1.85. (Figure 2D,E, Table S1). Immunofluorescence staining demonstrated sufficient expressions of glucagon and insulin in the isolated islets (Figure 2F,G).

### 3.5. Functional Analysis of Mitochondrial Oxidation

**Figure 3:**
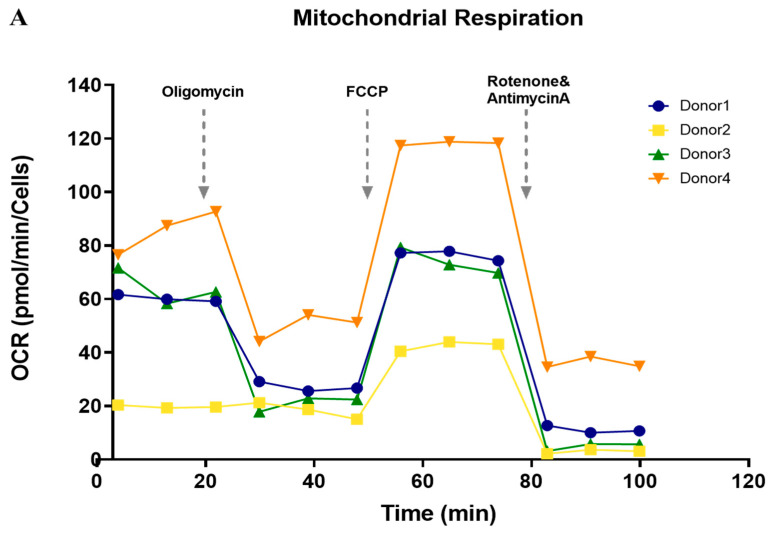

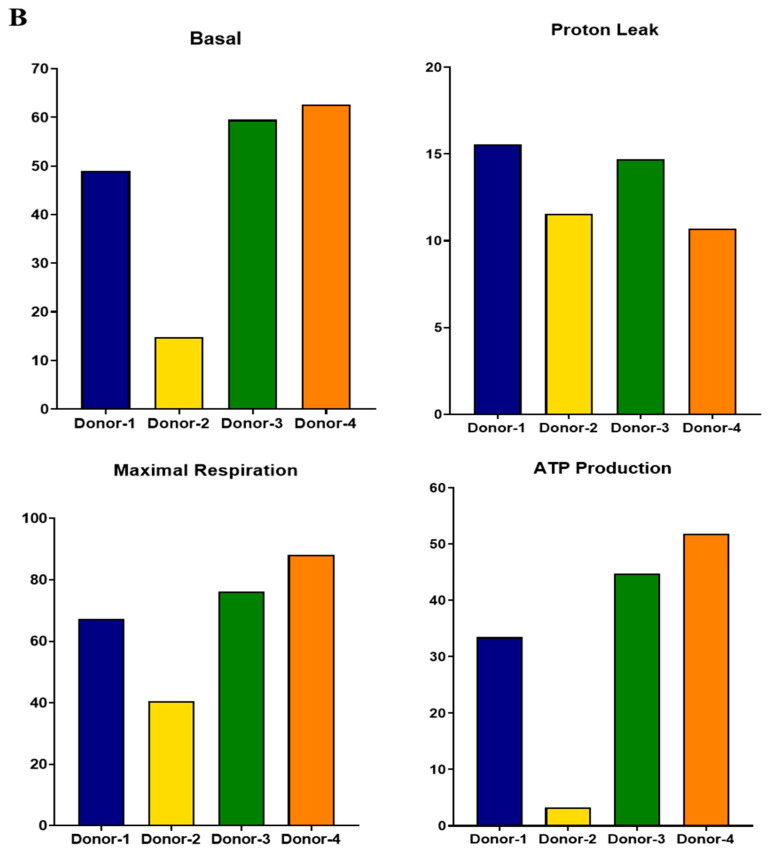
Mitochondrial function of islet cells isolated from partial pancreas tissue. (**A**) Observation of changes in oxygen consumption rate (OCR) during the respiration of mitochondria in isolated islet cells and (**B**) basal respiration, proton leak, maximal respiration, and ATP production (*n* = 4).

We performed a seahorse analysis on islets to observe whether OCRs for mitochondrial respiration were maintained in isolated islets. Good-quality islets (donor 1, 3, and 4 in Figure 3A) demonstrated a pattern similar to that of normal viable cells, whereas some islets (donor 2 in Figure 3A) showed reduced OCR levels, including basal OCR, proton leakage, maximal respiration, and ATP production (Table S1). These findings suggest that mitochondrial function was generally well-maintained in isolated islets.

### 3.6. Cryopreservation of Islet Cells Isolated from Partial Pancreas Tissue

**Figure 4:**
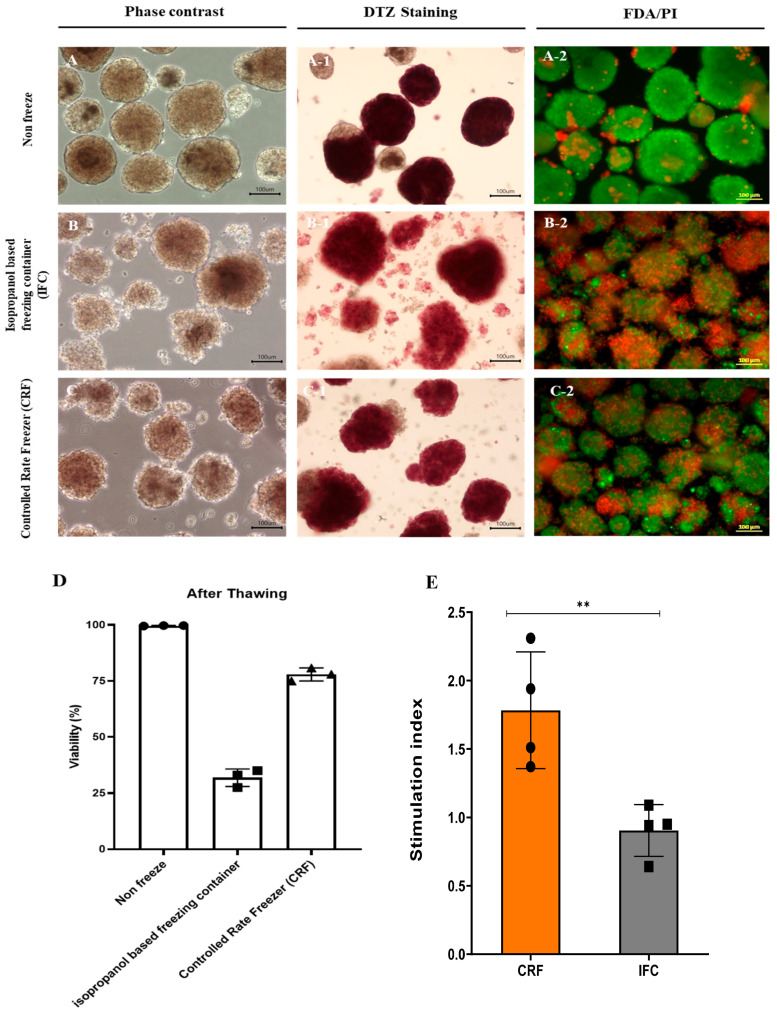

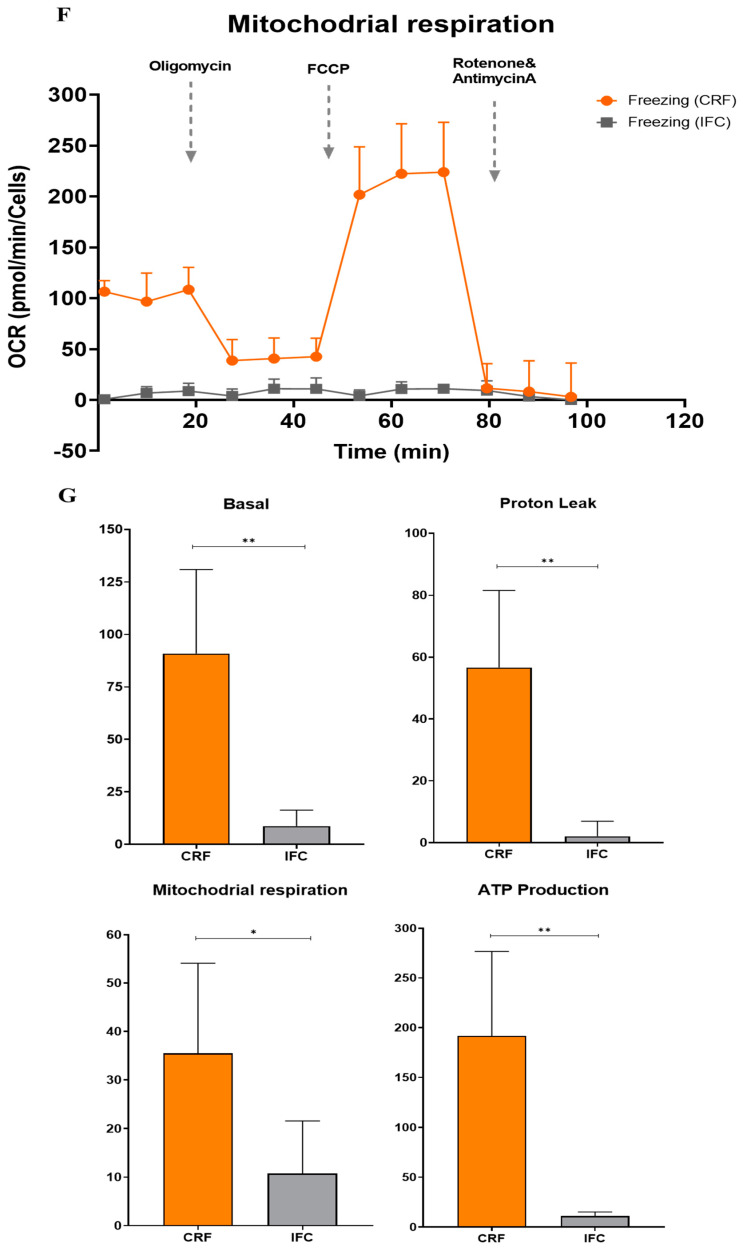
Cryopreservation of pancreatic islet cells isolated from partial pancreatic tissue. (**A**) Morphology and viability of islet cells cultured without cryopreservation and after freezing/thawing using (**A_1**) Dithizone staining of islet cells cultured without freezing/thawing (**A_2**) FDA/PI staining of islet cells cultured without freezing/thawing (**B**) an isopropanol-based freezing container (**B_1**) Dithizone staining of islet cells after freezing/thawing in isopropanol-based freezing containers (**B_2**) FDA/PI staining of islet cells after freezing/thawing in isopropanol-based freezing containers and (**C**) a controlled-rate freezer (CRF); scale bar, 100 µm. (**C_1**) Dithizone staining of islet cells freezing/thawing in a controlled-rate freezer (CRF) (**C_2**) FDA/PI staining of islet cells freezing/thawing in a controlled-rate freezer (**D**) Quantitative analysis of cell viability after thawing, using FDA/PI staining (*n* = 3). Analysis of mitochondrial function after freezing. (**E**) GSIS-based stimulation index after thawing of islet cells. (**F**) Changes in oxygen consumption rate (OCR) during mitochondrial respiration in isolated islet cells after thawing and their (**G**) basal respiration, proton leakage, maximal respiration, and ATP production levels (*n* = 3). *: *p* < 0.05, **: *p* < 0.01.

The successful cryopreservation of cultured islets is a prerequisite for islet transplantations and future research. We investigated the feasibility of the cryopreservation of islets isolated from partial pancreas tissue. We compared the conventional protocol that uses an isopropanol-based freezing container (IFC) with the controlled-rate freezer (CRF) protocol. Samples in both groups contained 1500 IEQ and were frozen in the same cryopreservation medium. After thawing, samples frozen using the conventional protocol yielded 1320 IEQ, while the CRF protocol produced 1405 IEQ (88% vs. 93.02%) (Table 3). In addition, DTZ staining revealed that samples frozen with the conventional protocol exhibited more islets dissociating into single cells compared to samples from the non-frozen group and the CRF group (Figure 4). After thawing, cell viability was confirmed via FDA/PI staining (Figure 4A–C). Samples in the non-frozen and the CRF groups demonstrated good viability compared to the IFC group. Based on FDA/PI staining, samples in the non-frozen, IFC, and CRF groups exhibited viabilities of 99.63 ± 0.09%, 31.83 ± 3.17%, and 77.93 ± 2.36%, respectively. In addition, the CRF group showed better results in a mitochondrial analysis after thawing than the IFC group, as well as a higher SI as per the GSIS analysis (Figure 4D–G). These data indicate that islet cells isolated from partially resected pancreas tissues demonstrated high viability after cryopreservation and thawing using the CRF compared to the IFC freezing protocol.

### 3.7. In Vivo Function of Islet Cells Isolated from the Partially Resected Pancreas

**Figure 5:**
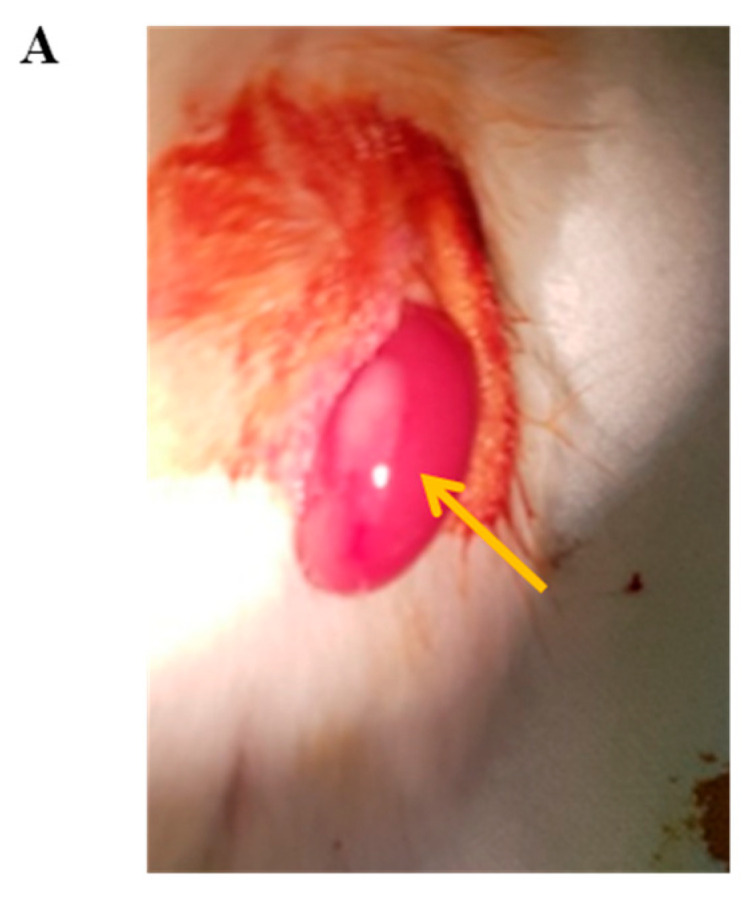

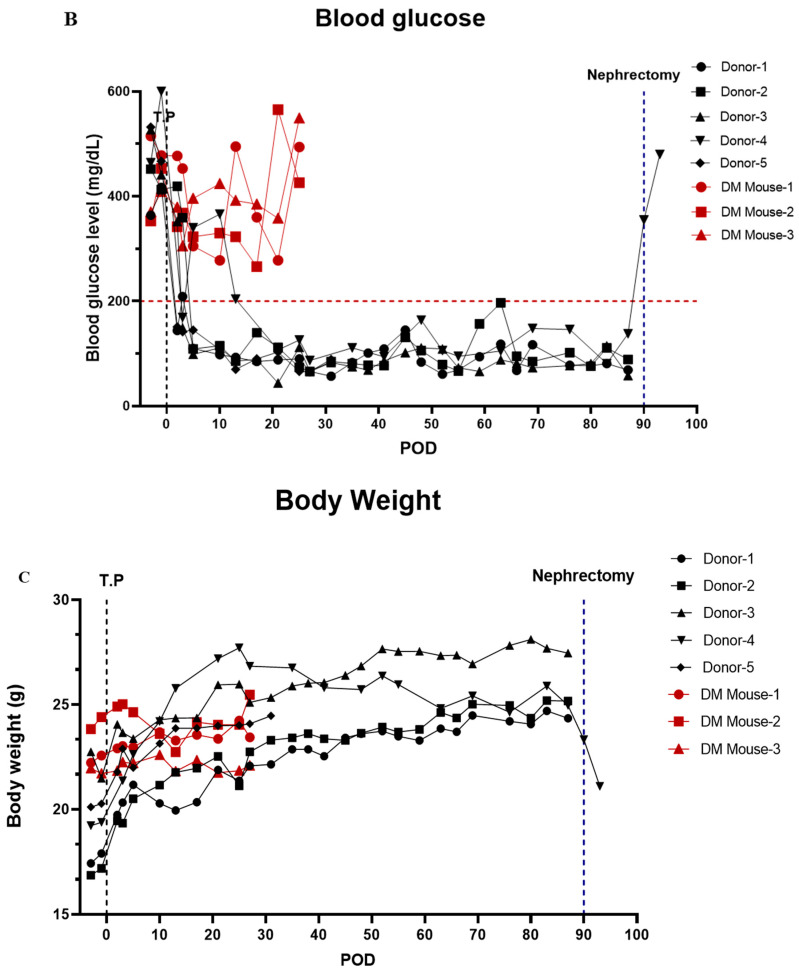

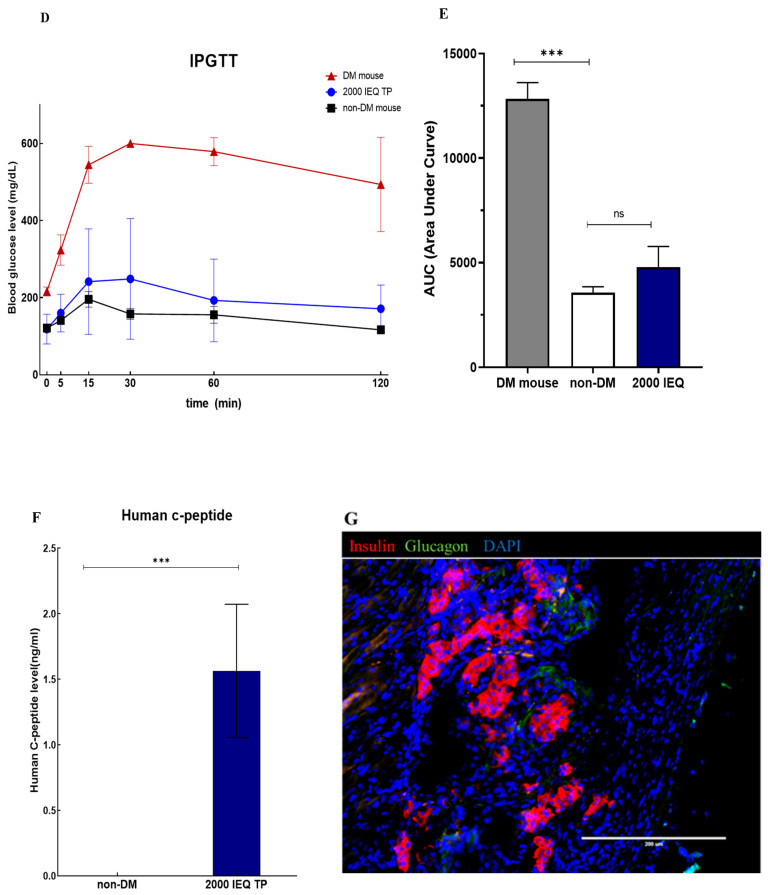
Evaluation of the in vivo function of islet cells isolated from partial pancreas tissue. (**A**) Transplantation of 2000 islet equivalents (IEQ) of islet cells isolated from pancreatic tissue into the kidney membrane of diabetic mice. Arrow, islet cell transplanted into the kidney membrane. Observations of changes in (**B**) blood glucose levels and (**C**) body weight (*n* = 8). (**D**) Intraperitoneal glucose tolerance test (*n* = 9). (**E**) Area under the curve based on an intraperitoneal glucose tolerance test (IPGTT) (*n* = 9) *** *p <* 0.0001, ns (Not significant) (**F**) Human c-peptide levels 90 days after islet cell transplantation (*n* = 3). (**G**) Immunofluorescence staining of the expression of glucagon and insulin 40 d after islet kidney transplantation. (Scale bar: 200 μm) *** *p <* 0.0001.

We also tested whether islet cells isolated from partially resected pancreas tissue can regulate diabetic hyperglycemia in vivo. When transplanted into the kidney capsules of immunocompromised STZ-induced diabetic mice, hyperglycemia was rapidly reversed to normal blood glucose levels (Figure 5, Table S1). After the transplantation of the islet cells, the blood glucose levels of the mice averaged < 200 mg/dL, and normal blood glucose levels were maintained (Figure 5B). In addition, the weight of the mice gradually increased after islet cell transplantation (Figure 5C). Hyperglycemia re-emerged after the removal of the kidney hosting the transplanted islets. In addition, the IPGTT showed similar results when conducted on diabetic mice transplanted with islets and normal mice, with human c-peptide being detected in the serum even after 90 days (Figure 5D–F). The expression levels of glucagon and insulin were confirmed by immunofluorescence staining of the islet kidney grafts after 40 days (Figure 5G).

## 4. Discussion

Access to human pancreatic islets is vital to ensure progress in the pursuit of novel therapeutic strategies for diabetes. However, human beta-cell research is constrained by the limited availability of human pancreatic islets from living or cadaveric donors. Due to the challenge of obtaining human islets for diabetic research, researchers have used the laser-capture microdissection (LCM) of pancreatic tissue to study the pathogenesis of islets in diabetes. However, while LCM enables the collection of gene expression data for analysis, it does not provide sufficient numbers of living cells for functional and proteome studies. In this context, the acquisition of living islet cells from discarded pancreatic tissue after surgical resection to treat benign pancreatic diseases can be an effective tool for researchers studying diabetes. Relatively few studies have reported on the process and outcomes of isolating islets from surgical specimens, and outcomes regarding the quality of the islets derived from surgical specimens are rarely reported. Bötticher et al. reported the isolation of islets from 24 partially pancreatectomized patients using various methods with brief functional analyses [10]. In this study, we have confirmed that high-quality human islets can be prepared from tissues that are discarded after surgery. Furthermore, we have evaluated the cell viability, functionality, mitochondrial features, nucleic acid extraction, cryopreservation, and in vivo qualities of the isolated cells to confirm that the isolated human islet material is viable for various diabetes studies. Unfortunately, we were able to evaluate the quality of islets in only some of the samples because we realized the importance of systematic quality assays only recently.

The patient cohort included 42.2% type 2 diabetes patients, and islets from these patients were harvested similarly to those without diabetes. The quality of islets derived from donors with diabetes was comparable to those from patients without diabetes.

Previous studies using pancreas tissue procured from brain-dead donors have demonstrated that several factors, such as donor age, BMI, pancreas size, pancreatic quality, enzymes, and digestion duration, are associated with islet yields [14,15,16,17]. We investigated the factors influencing the efficiency of pancreas islet isolation from surgical specimens and compared them with the factors that affect the efficiency of pancreas islet isolation from brain-dead donors.

After purification, the islet yield was a total of 59,593 ± 56,651 IEQ with 71.5 ± 21% purity, whereas, in our previous report, the cadaveric pancreases yielded a total of 130,600 ± 140,200 IEQ with 54 ± 31% purity [7]. We obtained an average of 2546 IEQ/g of the digested pancreas, which compares favorably to the median of 1676 IEQ/g of the cadaveric pancreas reported in our previous study. Similarly, Wang et al. reported yields of approximately 2684 IEQ/g [18] using a cadaveric pancreas. Bötticher et al. reported close to 500 IEQ/g of pancreatic tissue when they directly injected collagenase or Liberase into pancreas parenchyma obtained from surgical specimens to distend the pancreas [10]. We used the ductal injection of collagenase similar to the protocol used for isolation from cadaveric donors) while preserving the pancreatic duct in the pancreas tissue. Thus, our islet isolation method allowed us to obtain a higher number of islets from discarded partial pancreatic tissue compared with the method used by Bötticher et al [10].

From our analysis of factors influencing islet yield, we observed significant differences in islet isolation outcomes between patients with and without diabetes. Our results showed that the islet yield from diabetic pancreases before purification was significantly lower than that from non-diabetic pancreases. These results were consistent with findings from previous studies showing a reduction in β-cell mass in diabetic pancreases [19,20,21]. Another factor significantly associated with better isolation outcomes in our study was the pancreas region used. The regions of the adult pancreas are anatomically divided into the head, body, and tail. Regional heterogeneities in the histology of islets have been well studied in rodents, with largely similar observations that the density of β-cell masses in the body and tail regions is higher than in the head region [22,23]. A previous study reported that in humans, similar to rodents, the tail region contained > a 2-fold higher islet distribution than the head and body region. The regional difference in islet density is reflected in the yield of isolated human islets (normalized to regional pancreas weight) in that the yield was > 2-fold higher in the body and tail region compared to the head region [24]. In agreement with previous studies, we confirmed that the tail portion of the pancreas contains a higher number of islets (3000.51 ± 2773.16 IEQ/g pancreas). While donor gender, pancreas status, and digestion time were significant factors in the analysis of islet isolation factors from the cadaveric pancreases, these factors were not significant in the current study [7].

Since partially resected pancreatic tissues can degrade due to warm ischemia and other surgical or mechanical injuries, it is critical to guarantee the quality of islet preparations procured from discarded partial pancreas surgical specimens. To evaluate whether these islets could be used for various research purposes, we examined the morphology and functionality of islets in several samples. Isolated islets from partially resected pancreases maintained a good three-dimensional structure, displayed normal insulin release in response to high glucose stimulation in vitro (SI 3.85 ± 1.85), and restored euglycemia after transplantation into STZ-induced diabetic nude mice.

The acquisition of good-quality RNA samples from pancreatic tissue is difficult due to the presence of auto-digestion enzymes in the pancreas. We checked the quality of RNA extracted from cells to determine whether the quality was sufficient for use in various molecular studies. The RNA obtained following islet isolation was of high quality (as revealed by high RIN values) and had a high 260/280 nm (>2) ratio. These requirements are necessary to increase the reliability of diverse molecular analyses. In addition, we demonstrated that key transcriptional factors expressed in islet cells could be assessed using RT-PCR. We have used these islets for collaborative work with the International Epigenomic Study for Diabetes Research [25].

Recently, research on mitochondria has attracted attention with respect to cell function and development. Our data show that the mitochondrial function of the islets harvested from surgical specimens was well maintained. A mutational analysis of the mitochondrial DNA in representative islets indicated that there were no abnormalities, indicating that mitochondrial studies are also feasible using pancreatic islets derived from surgical specimens.

Cryopreservation is thought to be an ideal method for the long-term storage of human pancreatic islets, and many investigations on the use of islet cryopreservation techniques have been performed [26,27,28,29,30,31,32,33,34]. The major disadvantage of cryopreservation is the deterioration of the number and function of islets after thawing [35]. Although we obtained a high islet viability of close to 80% using the CRF method, the sample size was not large enough to draw a definitive conclusion.

Despite the originality of our research, this study has some limitations. First, we could not perform quality tests on all of the samples because the importance of the detailed quality assurance of islets derived from surgical specimens in the early period of isolation only became evident later in the study. Once we realized the importance of ensuring islet quality during isolation, we checked the quality of the islets in a few cases. Although only a small number of cases were analyzed, most of the procedures involved in the surgery and islet isolation were similar throughout the experiments, suggesting that these data likely reflect the features of most islet samples. Further research with a larger number of samples is needed to draw more robust conclusions. Second, the researchers responsible for islet isolation changed once, and it is possible that this affected the analyses of the isolation outcomes, despite adherence to the protocol where possible. Third, we could not compare our protocol with other currently existing protocols. Instead, we compared our results with those previously published. There are only two reports on the isolation of islets from pancreatectomy specimens after resection. However, they did not report on the quality of islets in detail. Gregor Bötticher et al. reported yields of an average of ~500 islets per gram of pancreatic tissue, with great variation. They achieved > 90% islet purity by first staining with dithizone during tissue processing and then handpicking them 24 h after isolation. They tested for 25 mM glucose-stimulated insulin secretion and recorded insulin secretion comparable to that of islets obtained from cadaveric islet isolation; they used islets for the study of specific protein or gene expression. Their protocol was similar to ours except for the cannulation of the pancreatic duct for the infusion of collagenase. Michele Solimena et al. retrieved islet specimens by LCM from snap-frozen surgical specimens. They did not perform any functional assays except a genetic study.

## 5. Conclusions

Our findings suggest that islet cells isolated from pancreases discarded after partial surgical resection exhibited good quality islet morphology, function, cellular composition, and recovery after cryopreservation. This method can be used as a valuable source of cells for islet cell research on the treatment of diabetes.

## Figures and Tables

**Table 1:** Donor characteristics and outcomes of islet isolation.

Characteristic	Value
Donor (*n*)	82
Age (years)	54.6 ± 15.1
Sex	Female: 52.4%, Male: 47.6%
BMI (kg/m^2^)	25.5 ± 8.6
Disease for resection	NET 20%, IPMN 16.7%, SPN 10%, SPT 10%, MCN 10%, Other disease 33.7%
Diabetes in underlying disease	No: 57.8%, Yes: 42.2%
Specimen location in pancreas	Head: 26.8%, Body and Tail: 73.2%
Time from operation room to laboratory (min)	31.1 ± 7.9
Specimen size (g)	23.4 ± 10.6
Enzyme for digestion	Collagenase P: 43.9%, Liberase MTF/CT: 56.1%
Digestion time (min)	10.5 ± 5.8
Before purification islet IEQ	124,006 ± 88,959
After purification islet IEQ	59,593 ± 56,651
Purity (%, DTZ staining (+))	71.5 ± 20.9

**Table 2:** Multivariate analysis of factors affecting pancreas islet isolation outcome.

	Outcome: IEQ before Purification per Tissue Weight (*n* = 82) †				Outcome: IEQ after Purification per Tissue Weight (*n* = 82) †			
			Multivariable				Multivariable	
	Mean	SD	SE	*p*-Value	Mean	SD	SE	*p*-Value
**Sex**								
M	4617.67	3461.01			2061.48	1525.37		
F	6306.61	4111.94			2990.06	3141.08		
**Age**								
<40	6163.71	3138.68			2134.05	1750.32		
≥40	5356.61	4073.58			2657.84	2705.37		
**DM**								
0	6588.86	4205.94			3088.35	2848.05		
1	3712.82	2656.2	0.16319	0.0009	1655.95	1701.14	0.224	0.0046
**BMI**								
<23	5690.83	4542.5			2776.7	3242.78		
≥23	5442	3576.69			2443.24	2142.74		
**Specimen location** **in pancreas**								
Head	3063.71	2589.58			1115.59	869.95		
Body and tail	6364.14	3982.25	0.18417	0.0096	3000.51	2773.16	0.251	0.0156
**Enzyme type**								
Liberase	4695.78	3550.12			2005.06	1815.83		
Collagenase	6582.13	4103.68			3264.03	3140.41		
**Digestion time**								
<10	4038.22	3542.01			2413.42	2327.99		
≥10	6785.75	3789.76	0.15404	<0.0001	2759.1	2718.19		

† Log transformation of outcome variable was applied.

**Table 3:** Islet equivalent (IEQ) of islet cells after cryopreservation and thawing.

	Before Freezing (IEQ)	After Freezing (IEQ)	Recovery Rate (%)
**Not frozen**	1500	1504	100.3
**Isopropanol-based freezing container (IFC)**	1500	1320	88
**Controlled-rate freezer (CRF)**	1500	1405	93.7

## Data Availability

Not applicable.

## Supplementary Materials

The following supporting information can be downloaded at: https://www.mdpi.com/article/10.3390/cells11152335/s1, Table S1: Donor information on islets used in the functional assay; Figure S1: (**A**) Correlation between diabetes patients and islet cell isolation efficiency (*n* = 19). (**B**) Correlation between pancreatic weight and islet cell isolation efficiency (*n* = 82). (**C**) Islet cell isolation efficiency by pancreatic region (*n* = 82).
