# Supplementary material for: The Outcomes and Quality of Pancreatic Islet Cells Isolated from Surgical Specimens for Research on Diabetes Mellitus
Source: Cells. 2022 Jul 29;11(15):2335. doi: 10.3390/cells11152335 (PMC9367344; doi:10.3390/cells11152335)
Supplement: Supplementary file 1 [file cells-11-02335-s001.zip › cells-1803605-supplementary.pdf]

Supplementary Information

Table S1. Donor information on islet used functional assay

A. Mitochondrial function

Characteristic	Patient 1	Patient 2	Patient 3	Patient 4
Age (years)	31	26	57	39
Sex	Female	Female	Male	Female
BMI	21.89	18.83	22.89	23.73
Disease for resection	MCN	MCN	SCA	SPN
Diabetes in underlying disease	No	No	No	No
Specimen location in Pancreas	Tail	Tail	Tail	Tail
Specimen size (g)	21.55	27.61	24.86	21.56

B. GSIS

Characteristic	Patient 1	Patient 2	Patient 3	Patient 4
Age (years)	54	61	57	39
Sex	Male	Female	Male	Female
BMI	26.5	20.84	22.89	23.73
Disease for resection	NET	PanIN	SCA	SPN
Diabetes in underlying disease	No	No	No	No
Specimen location in Pancreas	Tail	Tail	Tail	Tail
Specimen size (g)	46	21.19	24.86	21.56

C. Islet transplantation

Characteristic	Patient 1	Patient 2	Patient 3	Patient 4	Patient 5
Age (years)	54	61	31	26	39
Sex	Male	Female	Female	Female	Female
BMI	26.5	20.84	21.89	18.83	23.73
Disease for resection	NET	PanIN	MCN	MCN	SPN
Diabetes in underlying disease	No	No	No	No	No
Specimen location in Pancreas	Tail	Tail	Tail	Tail	Tail
Specimen size (g)	46	21.19	21.55	27.61	21.56

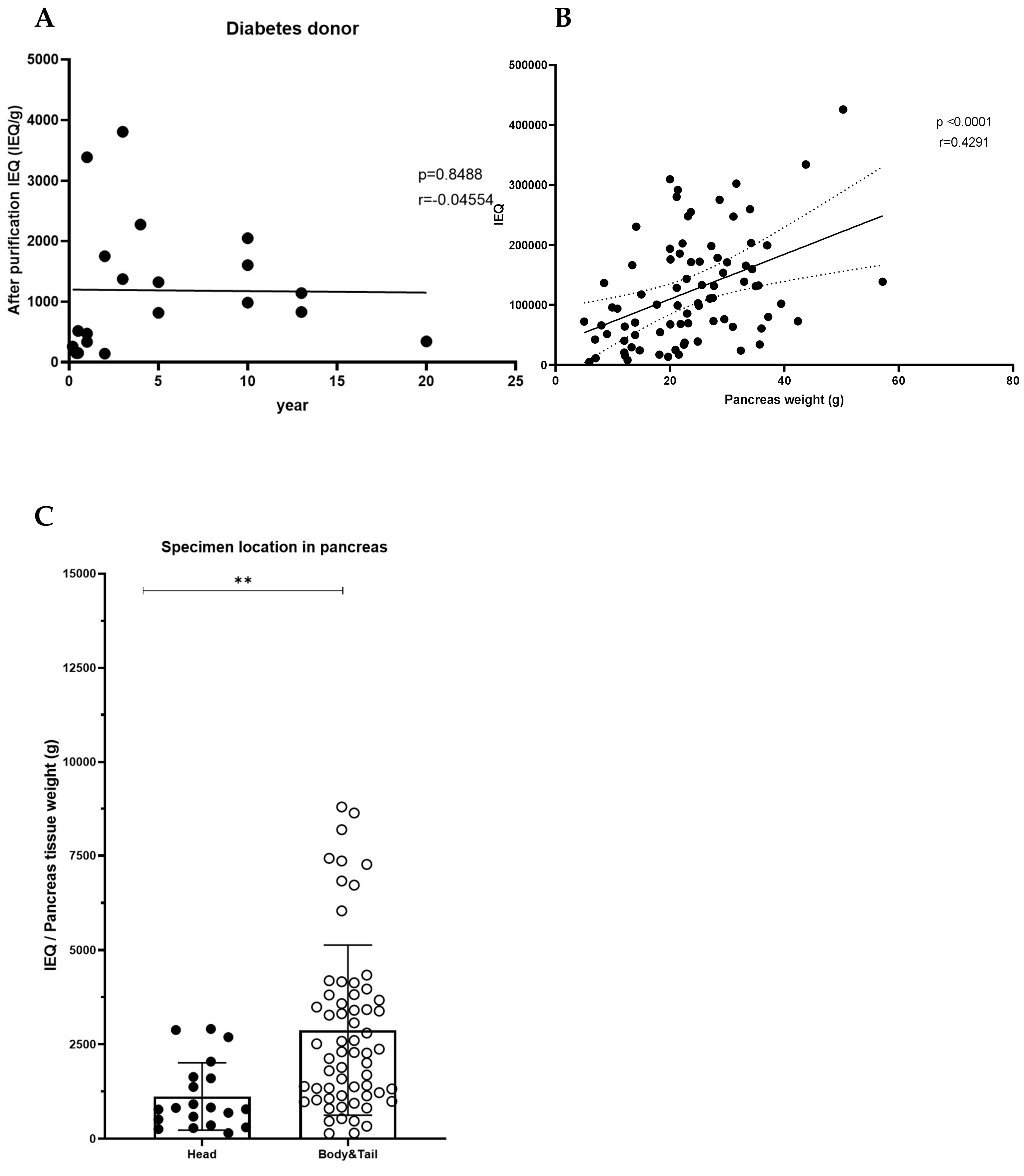

Figure S1. (**A**) Correlation between diabetes patients and islet cell isolation efficiency (n=19). (**B**) Correlation between pancreatic weight and islet cell isolation efficiency (n=82). (**C**) Islet cell isolation efficiency by pancreatic region (n=82) ** $p < 0.01$.
